# Editorial: Relationship between subjective well-being and mental disorders across the lifespan

**DOI:** 10.3389/fpsyg.2023.1268287

**Published:** 2023-08-15

**Authors:** Natalia Martín-María, Elvira Lara, Anna K. Forsman

**Affiliations:** ^1^Department of Biological and Health Psychology, Faculty of Psychology, Universidad Autónoma de Madrid, Madrid, Spain; ^2^Department of Personality, Assessment, and Clinical Psychology, Faculty of Psychology, Universidad Complutense de Madrid, Madrid, Spain; ^3^Department of Health Sciences, Faculty of Education and Welfare Studies, Åbo Akademi University, Vaasa, Finland

**Keywords:** positive mental health, positive affect, psychopathology, aging, ill-health

The Frontiers Research Topic entitled: “*Relationship between subjective well-being and mental disorders across the lifespan*” is aimed at analyzing the associations between these two concepts throughout the life cycle.

Subjective wellbeing is a high priority on the global public health agenda. The comprehensive mental health action plan 2013-2030 of the World Health Organization (WHO, 2021) indicates as general objectives to protect and promote the wellbeing of all citizens, to prevent mental disorders, and to improve care for people with mental illnesses. Subjective wellbeing has been shown to positively affect health status (Steptoe et al., 2015), whereas mental problems is one of the leading causes of unhappiness (Helliwell et al., 2013). Given the unprecedented situation we lived after the disruption of our daily living routines, it is important to study whether the COVID-19 pandemic has affected subjected wellbeing and mental health, especially among vulnerable groups (e.g., young people, and/or older people with chronic diseases).

Seven articles were included in this Research Topic collection, comprising different sample populations and an age range that varied from 12 to 118 years.

Using a differential game methodology, Bai and Ma examined how to protect the mental health of the isolated population of China due to the COVID-19 pandemic. This method implies the mathematical study of situations of conflict of interest. In this article, game theory considers the individual and the government's behavior, and studies their optimization strategies. To effectively control the spread of the disease, China strictly implemented the general policy of “dynamic zero clearance”. To alleviate the psychological problems of the isolated masses three measures were analyzed: self-regulation, government psychological counseling, and social force psychological counseling. Which method has more prominent psychological effects on residents, and which one achieved the fastest way for the isolated population to go from depression to wellbeing was the focus of this article. The authors concluded that both the government and social forces were important to obtain psychological benefits, yet they should work together to give an adequate counseling for isolated people. Sim and Im also analyzed the impact of COVID-19 on the mental health of Korean college students. They combined quantitative and qualitative research methods to study how the participants coped with the pandemic and if they achieved any positive change or growth. Participants reported COVID-19-related concerns and difficulties that further increased depression and anxiety levels significantly. Coping flexibility moderated the impact of COVID-19-related concerns and difficulties on depression and anxiety; however, it amplified the impact of such disruptive emotions. On the contrary, a sense of community reduced the consequences of these overwhelming worries on them.

Younger people could experience lower mental wellbeing and body appreciation compared to older people. Regarding the implications of person-centered evaluation of positive body image, Zhang et al. observed in a cross-sectional study using a convenience sampling that more than half of Chinese nursing students were disappointed with their body figure. The stress, anxiety, and depression of nursing students were significantly and negatively correlated with body appreciation and self-concept clarity. They highlighted the fact that negative social media exposure has harmful influence on body image, emotions, and living lifestyles, especially on young women.

Mental health literacy (MHL) refers to knowledge and beliefs related to mental illness, and it is an important determinant of mental health. Considering that there are very few scales for assessing positive MHL in China, Liu et al. explored the psychometric characteristics of a Chinese version of the positive mental health literary scale (MHPK-10-C) among Chinese adolescents. The authors found sound reliability and validity properties and suggested that can be used to measure positive MHL in Chinese adolescents.

Furthering the evidence from studies focusing on the young population, Block et al. explored the importance of meaningful relationships for mental health in both a clinical and a convenience community sample that that included participants 18 years of age or older. Meaningful relationships are essential for the functioning of all human beings. Contrary to expectations, they observed that clinical participants did not present a deficit in fulfilling relationships. Most people reported a well-functioning network of meaningful, high-quality relationships. Meaningful relationships could be a crucial part of risk evaluation when treating a new patient.

Using a large-scale nationally representative dataset (more than 25,000 of participants), the Chinese General Social Survey, Li et al. examined the relationship and cohort variations between subjective wellbeing and depressive disorders. In line with previous literature (Cummins, 2013), they noticed that higher levels of subjective wellbeing decreased perceived depression. Regarding different age groups, subjective wellbeing values were lower in the 20–30 and 30–40 age groups, and then rose considerably in the 40–50 age group. The negative relationship between age and subjective wellbeing showed that with age increasing, the impact of happiness on reducing perceived depression tends to be stronger. In consequence, subjective wellbeing takes a relevant role in supressing perceived depression in older population.

Finally, Karwetzky et al. analyzed the correlations and differences in life satisfaction throughout the life cycle based on a cross-sectional survey with almost 1600 participants aged 12–94. The authors observed that life satisfaction correlated negatively with poor health and financial worries, and positively with partnership, grandchildren, and religiosity. However, the inverse relationship with poor health was more important in younger than in older participants, while the inverse association with financial worries was stronger in late midlife (50–69 years). Moreover, their results showed that while men displayed a U-shape trend in the relationship between age and life satisfaction, with its lowest point between 30 and 49 years of age, women' happiness raised stepwise with age. Therefore, people adjust or even grow beyond their perceptions of life satisfaction over time. Neurobiological processes of adaptation and personal growth were proposed as possible determinants.

In conclusion, this Research Topic collection sheds light on the potential of subjective wellbeing in improving mental health across the lifespan. Most studies used a cross-sectional design to analyse a relationship between variables. In consequence, there is a need for more longitudinal and intervention studies focused on how promoting individuals' subjective wellbeing may help reduce mental disorders, comorbidity, or even mortality.

## Author contributions

NM-M: Writing—original draft. EL: Writing—review and editing. AF: Writing—review and editing.
